# Tracking decarbonization of multilateral development banks’ electricity generation investments

**DOI:** 10.1016/j.crsus.2025.100584

**Published:** 2025-12-26

**Authors:** Florian Egli, Clemens-Maria Lehofer, Nadine Palmowski, Tim Büthe, Bjarne Steffen, Tobias S. Schmidt

**Affiliations:** 1 School of Social Sciences and Technology, Technical University of Munich, Munich, Germany; 2 Energy and Technology Policy Group, ETH Zurich, Zurich, Switzerland; 3 Munich School of Politics and Public Policy (HfP), School of Social Sciences and Technology and School of Management, Technical University of Munich, Munich, Germany; 4 Sanford School of Public Policy, Duke University, Durham, NC, USA; 5 Climate Finance and Policy Group, ETH Zurich, Zurich, Switzerland; 6 Albert Einstein School of Public Policy, ETH Zurich, 8092 Zurich, Switzerland; 7 School of Management, Technical University of Munich, Munich, Germany

**Keywords:** international financial institution, development, energy transition

## Abstract

Multilateral development banks’ (MDBs) commitment to the Paris Agreement (PA) was expected to induce a shift from fossil fuel-based to sustainable energy sources in the Global South. However, we lack a comprehensive analysis of their electricity generation portfolios and internal policies since then. This paper presents two new datasets on 1,230 electricity generation investments and 215 decarbonization policies adopted by all MDBs from 2006 to 2020. We find a continued decline in fossil fuel investment since the PA but no change in pace. The volume of investment in renewables (including hydropower) has not increased enough to compensate for the phaseout, resulting in a downward trend in MDB electricity generation investments over time. The number of renewable projects funded by MDBs, however, has substantially grown. These findings raise concerns about MDBs’ ability to scale up clean electricity investments, particularly in low-income countries, where the energy investment gap continues to grow.

## Highlights

• MDB investments in electricity generation declined after the Paris Agreement
• MDBs decarbonize their portfolios, but the Paris Agreement did not accelerate this trend
• Newly founded MDBs started out almost fully decarbonized
• Low-income countries receive comparatively little investment

## Science for society

Addressing climate change requires the rapid decarbonization of the electricity sector. In emerging and developing countries, where electricity demand is rising, this requires large infrastructure investments. Due to limited public resources and unfavorable risk assessments by private investors, infrastructure projects in many of these countries rely on the involvement of multilateral development banks (MDBs). However, it remains unclear to what extent MDBs deliver on supporting the transition to renewable energy technologies since the Paris Agreement (PA). Here, we present two new datasets on MDB investment in electricity generation and internal climate policies, covering 10 years before and 5 years after the PA. We show that decarbonization has steadily advanced as MDBs have phased out fossil fuels. Yet, MDBs were unable to increase their renewable investments fast enough, leading to an overall decline in electricity generation investments despite the growing need.

## Graphical abstract

**Figure:**
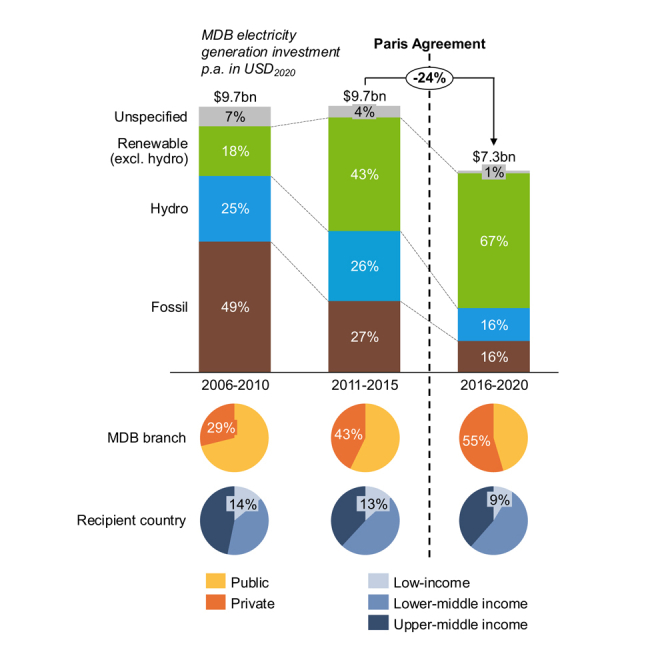

## Teaser

Egli et al. present new data on multilateral development banks, showing that the overall volume of electricity generation investment has not increased since the Paris Agreement. Fossil fuel investments declined, and renewable investment volumes were too small to reverse this trend. Their work shows the challenges in scaling up renewables, particularly in low-income countries with a growing need for clean energy.

## Introduction

Demand for electricity is projected to grow massively in the next decades, particularly in emerging and developing countries (EMDCs).^1^ To meet this demand in line with the Paris Agreement’s (PA) climate goals, massive investments in renewable electricity generation in EMDCs are required,^2^ in addition to phasing out fossil fuel investments.^3^ Yet, such investments in EMDCs globally fall short of what would be needed to reach climate goals.^4^^,^^5^

Multilateral Development Banks (MDBs) are key financiers of energy infrastructure in EMDCs and are therefore critical to global climate action.^6^^,^^7^^,^^8^^,^^9^ For instance, a G20 report found that between 2010 and 2021, MDB involvement in developing countries increased from 6% to 19% of total private investment in infrastructure.^10^ MDBs are often favored as vehicles for public climate finance, as they have technical expertise, can offer advantageous financing conditions to borrowers, and provide donor states with constrained public budgets a bigger lever in pursuing climate mitigation projects.^7^^,^^11^ Additionally, MDBs can amplify climate finance by mobilizing private capital, as first-movers and in a signaling role.^12^ Promoting clean power in the EMDCs, MDBs contribute to economic development as well as energy and climate goals agreed upon in the Sustainable Development Goals.

Previous research has shown that MDBs started decarbonizing their electricity generation portfolios already before the PA, albeit with large differences between MDBs and largely in their private sector branches only.^13^ While few cross-bank analyses have covered the period after the PA,^7^^,^^14^ most literature examining MDBs’ energy investment is limited to case studies of single banks or regions pre-PA.^8^^,^^15^^,^^16^ Although the MDBs have published joint annual climate finance reports since 2012 and have continued to make progress in harmonizing methodologies,^17^ these data are available in aggregated form only. This lack of project-level data impedes transparency and comparability across institutions in assessing the climate finance contributions of MDBs. Furthermore, previous analyses do not include two new MDBs that only started lending in 2016. It therefore remains unclear to what extent and which MDBs have made progress in decarbonizing their electricity generation portfolios since the PA.

In this article, we present two original datasets. The first is a comprehensive dataset of MDB electricity generation investments from 2006 to 2020, based on 1,230 MDB project reports. We subsume debt and loans, equity, grants, Islamic financial instruments, and performance-based instruments under the term “investment.” The other dataset provides detailed information about the MDBs’ 215 internal decarbonization policies adopted during those years. The novel data allow us to analyze MDBs’ electricity generation investment decisions (i.e., commitments) and internal governance after the PA, enabling a deeper understanding of the decarbonization progress, as well as the enabling factors and barriers.

## Results

Our analysis of MDB investment covers 15 years from 2006 to 2020. We do not extend our analysis beyond 2020 to avoid potential comparability issues, as MDB investments shifted dramatically to combat COVID-19 and its economic repercussions (see methods). We include electricity generation investments of all 10 MDBs, namely, the African Development Bank (AfDB), the Asian Development Bank (AsDB), the Asian Infrastructure and Investment Bank (AIIB), the Development Bank for Latin America (CAF), the European Bank for Reconstruction and Development (EBRD), the European Investment Bank (EIB), the Inter-American Development Bank (IDB), the Islamic Development Bank (IsDB), the New Development Bank (NDB), and the World Bank Group (WBG).

### Investment activity

**Figure 1:**
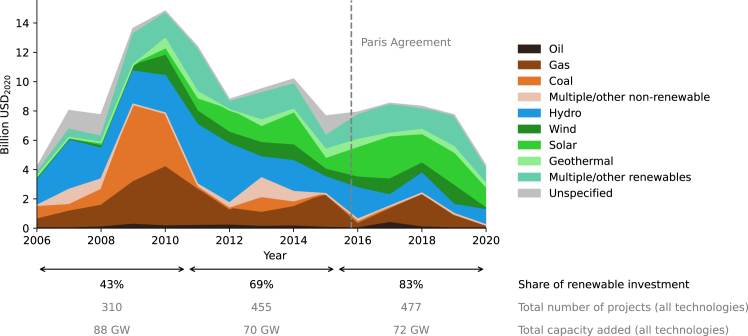
Yearly MDB investment by electricity generation technology Sub-figure numbers indicate the share of renewables, the total number of projects, and total capacity additions within the periods 2006–2010, 2011–2015, and 2016–2020. Data exclude guarantees and investments in high-income countries.

We observe two high-level trends in MDBs’ electricity generation investment. First, overall investment has declined from a peak of roughly USD_2020_ 15 billion/year in 2010 to USD_2020_ 4 billion in 2020 (Figure 1), a trend that started before and continued after the PA. Second, MDB electricity investments have almost fully decarbonized. MDBs increased their renewable financing from an average of 43% in 2006–2010 to 83% in 2016–2020. In 2016–2020, solar projects received the biggest share of investments. Among fossil fuels, investments in coal- and oil-based energy production have been almost entirely phased out; in 2016–2020, additional fossil fuel investments are primarily in natural gas-based technologies.

The decarbonization of the energy system requires a shift away from fossil fuels to renewables and a scale-up of renewables.^2^ Yet, we find the decarbonization of MDB investment portfolios to be limited to shifting investments from fossil to renewables. Investments in hydropower have declined since 2011, while the volume of other renewable energy investments has fluctuated, not exceeding USD_2020_ 3 billion/year per technology. While we focus on investment in this study, we also look at the number of projects and associated capacity additions. The number of projects increased substantially (see bottom of Figure 1), but the average project became smaller, leading to approximately constant capacity additions (even slightly declining from the first to the second period). As renewables experienced large cost declines in recent years,^18^ the same level of investment should result in larger capacity additions; however, the shift to smaller projects may pose a scaling challenge, which is discussed further along.

**Figure 2:**
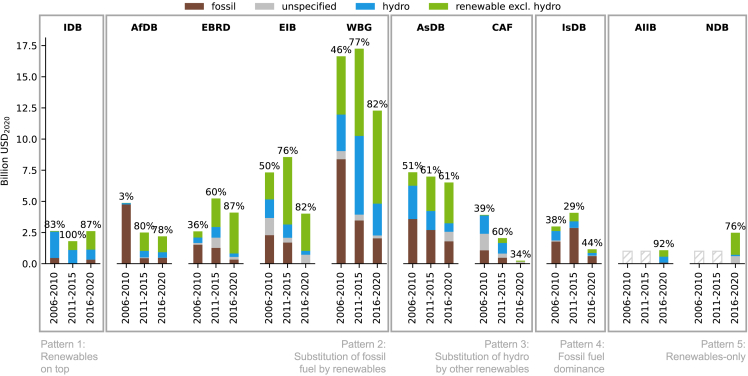
Total investment by electricity generation technology and individual MDBs per time period Data exclude guarantees and investments in high-income countries. Decarbonization ratio (share of hydro and renewable excluding hydro in total investments) is labeled above each bar.

Next, we investigate the heterogeneity among MDBs, which leads us to discern five patterns of decarbonization in Figure 2. Four of these match the patterns identified in the pre-PA analysis by Steffen and Schmidt.^13^ In the following, we discuss observed changes in the patterns and the allocation of MDBs to these patterns before the PA (based on Steffen and Schmidt^13^) and afterward (based on the novel dataset compiled in this paper). First, EBRD and EIB intensified their phase-out of fossil investments and transferred from “renewables on top” (pattern 1), where renewables dominate but fossil fuels persist, to “substitution of fossil fuels with renewables” (pattern 2), where fossil fuels are phased out simultaneously to rising renewables. Second, one MDB remains in the “fossil fuel dominance” pattern (#4) but stopped the growth of fossil fuel investments, which was observed before 2016. Third, all MDBs except IDB have seen a drop in their total electricity generation investments after the PA. Fourth, we identify a new pattern that did not exist before the PA: newly established MDBs (NDB and AIIB) show almost fully decarbonized portfolios from the start, introducing a new pattern of “renewables-only” (pattern 5). Founded in 2014 and 2015, respectively, these banks were launched at a time when renewable energy had already become viable and cheap. In other words, these newer MDBs did not have to overcome path dependencies to decarbonize, and contrary to some apprehensions at the time of their inauguration, these new MDBs did not replace the fossil fuel investments phased out by the other MDBs after the PA.

As a result, in the post-PA period (2016–2020), the MDBs that followed patterns 1–3 decarbonized about 73% of their portfolios on average. The IsDB (pattern 4) retains 52% of its investments in fossil fuel-based energy production, while the renewables-only new MDBs (pattern 5) invest 84% of their portfolio in renewable electricity generation on average.

**Figure 3:**
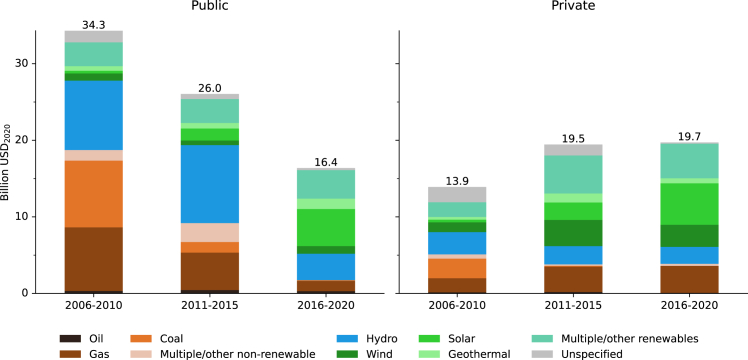
Total investment by electricity generation technology and MDB branch in respective time periods Data excludes guarantees and investments in high-income countries. Additionally, the figure excludes projects that do not clearly belong to one sector (e.g., public-private partnerships or undefined recipients).

MDBs lend to the public and the private sector via a bank’s public and private sector branches. While pre-PA research found that public branches decarbonized more slowly than private sector branches,^13^^,^^19^^,^^20^^,^^21^^,^^22^ we find that public sector branches have accelerated their fossil fuel phase-out after the PA, resulting in essentially the same portfolio composition as the private sector branches (see Figure 3). Public sector branches achieved this decarbonization by reducing fossil fuel (and hydropower) investments. Yet, the volume of these reductions outweighed the additions in non-hydro renewables, resulting in an overall decline in investments into electricity generation. At the same time, private branches did not substantially further reduce their (already low) fossil fuel investments, and in contrast to the public sector branches, they largely maintained their volume of electricity investment post-PA (2016–2020 vs. 2011–2015). Across both branches, we see a concentration of fossil fuel investments in gas after the PA, with a phase-out of most other fossil technologies.

### Decarbonization policies

Major changes such as decarbonization often reflect changes in MDB policies.^23^ Here, we describe the number and type of MDBs’ internal decarbonization policies over time, based on our policy dataset. Note that we do not causally link the enactment of internal policies to changes in investment portfolios, as establishing such a link is not straightforward^24^^,^^25^^,^^26^ and deserves a separate analysis in future research.

**Figure 4:**
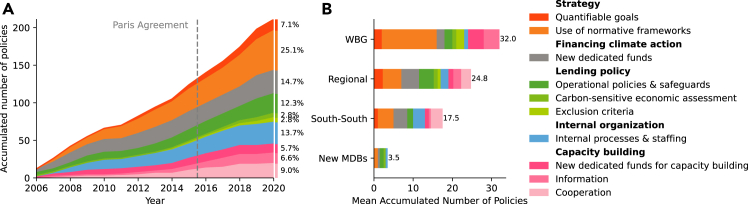
Energy and climate mitigation-related policies over time and by MDB groups (A) Accumulated number of policies per policy type over time. (B) Average number of policies implemented by MDB groups.

The dataset encodes all energy-related and climate mitigation-related policies officially adopted by one of the MDBs since 2006 and categorizes them into four groups: (1) formalized strategies, (2) financing climate action, (3) lending policy, and (4) internal organization and capacity building (see methods and Table S3). We conducted exploratory expert interviews (see Table S4) to develop the policy categories and discuss the comprehensiveness of the collected MDB policies. In Figure 4, we show the trends for all MDBs combined, as well as accumulated totals separately for the four groups: (1) WBG; (2) Regional MDBs, i.e., AfDB, AsDB, IDB, EIB, and EBRD; (3) established South-South MDBs, i.e., CAF and IsDB; and (4) the two most recently launched MDBs, i.e., NDB and AIIB.

We find that the total number of MDB policies in the field of climate and energy has been growing steadily since 2006. The PA does not appear to have resulted in a step-change in the preexisting trend of continuously increasing policy density, consistent with interpretations of international agreements as codifying rather than causing changes or trends.^27^ We find that normative frameworks, internal organization policies, and the establishment of new dedicated funds are the most frequently used policies. More recently, new policies like carbon-sensitive economic assessment have emerged. Across MDB groups, there is substantial policy variance, both in number and type (see Figures 4B and S1), over time. Numerically, the WBG leads in policy enactment, with 32 climate- or energy-related policies since 2006, followed by the Regional Banks, South-South banks, and finally, the New MDBs.

In terms of type, normative frameworks and new dedicated funds are prominently used across all banks. After the PA, the WBG introduced quantifiable goals and carbon-sensitive economic assessments for the first time, while the Regional Banks notably increased their cooperation policies post-PA. Among the policies that South-South banks introduced, we observe an increase in normative frameworks and a decline in capacity building.

The policy literature has developed a nuanced understanding of the stringency of policies, suggesting that codification of commitments under conducive conditions can bring about behavioral changes.^28^ Also, the MDB decarbonization policies coded in our dataset differ in stringency,^29^^,^^30^^,^^31^ as suggested in our exploratory expert interviews, too. For instance, “new dedicated funds” denote policies that specifically set aside capital for renewable investments and hence have a direct link to decarbonization. Other policies, such as internal staffing policies, may have large impacts on portfolio decarbonization but only in the long run and without a direct link. While establishing a causal link between these policies and decarbonization is beyond the scope of this paper, the information about differences in stringency will be critical for such analyses.

### Challenges to scaling investment in renewables

We have shown that MDBs have continued to decarbonize their portfolios post-PA by reducing their fossil fuel portfolio but failed to ramp up investments in renewable energies. We proceed to discuss two potential structural challenges to ramping up investment in renewables.

**Figure 5:**
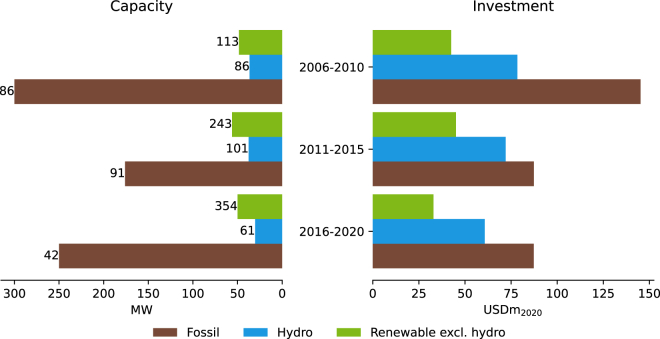
Median project size in terms of capacity (MW) and investment (USDm_2020_), per technology in respective time periods The number of projects per technology in the time period is labeled on the left of the bar. Data exclude guarantees and investments in high-income countries.

First, MDBs may struggle to ramp up renewable investment because of inherent differences between fossil fuel-based and non-hydro renewables-based electricity generation projects. As shown in Figure 5, the median capacity and the median investment of a renewable project consistently lie below fossil fuel projects (except for oil, as shown in Figure S2). As a result of the increasing share of renewables, the average project size over the entire portfolio—both measured in capacity and investments—declined over time (see Figure S3). While in 2006, renewable projects outnumbered fossil fuel projects by a factor of 3, this factor increased to 20 by 2020 (see Figure S3).

**Figure 6:**
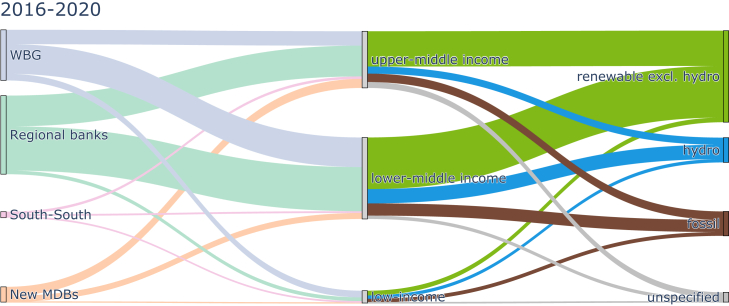
Total MDB investment flows toward countries grouped by income classification and electricity generation technologies, 2016–2020 Country classification follows the World Bank classifications and countries may move across groups over the observed time periods. Multi-country projects are excluded (see methods). Data excludes guarantees and investments in high-income countries. See Figure S5 for earlier periods.

Second, the geographical distribution of projects differs by technology, which may pose challenges when scaling renewables because investment destinations need to shift. The first imbalance occurs across income levels. By mandate, MDBs should invest in low- or middle-income countries. For 2016–2020, we find that most electricity generation investments flow to middle-income countries, as shown in Figure 6. Over time, we observe a disproportionate decline in investments and even more so in capacity additions in low-income countries. Capacity additions in low-income countries fell from 12 GW in 2006–2010 to only 1 GW in 2016–2020 (see Figure S4). While some of this change may be attributed to the progression of a few countries from low-income to lower-middle-income groups, some low-income countries see an >90% reduction in their investments for 2006–2010 to 2016–2020 (Ethiopia, Democratic Republic of the Congo, Guinea-Bissau, and Togo; see Table S1). Furthermore, even though a few other low-income countries have seen an increase in MDB electricity generation investment, the net change has been negative for low-income countries (see Table S1).

The income group disparity is also evident across technology groups. We observe the fullest decarbonization in middle-income countries with an average of 78% of renewables in 2016–2020 (see Figure 6). Conversely, we find that low-income and lower-middle-income countries are the primary recipients of the remaining fossil fuel investments. These two groups received two-thirds of fossil fuel investments in 2016–2020 (see Figure 6), while in earlier time periods, considerable fossil fuel investment went to (upper-)middle-income groups, too (compare with Figure S5A).

Overall, we observe that MDBs struggle to shift investments from fossil fuels to renewables without changing the country portfolio. Notably, on average 45% of the countries that received only fossil fuel investments in a given period received no MDB electricity generation investment in the subsequent period. This loss of investment persists. To illustrate, in the period of 2006–2010, 20 countries received only fossil fuel investments. Of these countries, only nine received MDB investment in the latest period of 2016–2020 (see Figure S6). In a few countries, MDBs successfully shifted their investment from fossil-only to renewables-only—Burundi, Djibouti, Indonesia, and Jamaica shifted from period 1 to 2; and Egypt, Ghana, Iraq, and Rwanda shifted from period 2 to 3. These observations indicate that there may be country-specific lock-ins, which make it easier for MDBs to decarbonize their portfolio by financing clean electricity generation assets in countries where they have not heavily financed fossil fuel infrastructure previously. This may be of concern to the countries that received large fossil fuel investments from MDBs in the past, such as Bangladesh, Egypt, or South Africa.

## Discussion

Our analysis of two novel datasets yields four key observations that are important for ongoing policy discourses. First, we observe that MDBs continued to decarbonize their portfolios after the PA at a similar rate to before. Decarbonization levels and speeds continue to differ across banks, with MDBs that were lagging before the PA remaining the least decarbonized and new MDBs being decarbonized from the start. At the same time, MDBs’ private branches, which were able to switch their fossil portfolio from coal to gas already before the PA, have made little further progress in decarbonizing. In contrast, public branches have reduced their fossil portfolio after the PA. Both branches struggle to ramp up renewables, leading to an overall decline in MDB electricity generation investments. Reasons for this decline remain to be investigated further and could entail analyses of MDB-internal processes, policy effects, or changing preferences from governments. Such research could leverage the compiled datasets for econometric analyses, for instance, testing whether policy changes coincide with structural breaks in investment.

Second, MDBs enacted various internal policy instruments related to energy and climate change. Similar to investment, we do not observe a major spike in policy adoption post-PA but a continuous upward trend among the established MDBs. The two new MDBs, with almost fully decarbonized portfolios, enacted the lowest number of climate and energy policies. They were founded at a time when clean energy assets had become mainstream, based on proven, reliable, and cost-competitive technologies. These findings point to two possible levers to decarbonize MDBs: introducing internal policies and incentives to overcome organizational and institutional path dependencies^32^^,^^33^ or founding new organizations at a time when desired decarbonization technologies are (about to be) mainstreamed, bringing about change through institutional layering.^34^^,^^35^ Both levers involve costs and benefits, which should be analyzed more systematically in future research, and likely need to be combined to achieve the required rate of change.

Third, despite an increase in the number of renewable projects, we observe that MDBs struggle to ramp up the investment volume in renewables. MDBs are designed to invest in large infrastructure projects. Hence, smaller ticket sizes of renewables may pose a challenge, as a higher number of small projects likely require more due diligence effort to achieve the same capacity additions. Owing to constrained public budgets, ramping up investment in renewables may be further challenging for South-South banks, because compared with fossil fuel investments, renewables still entail a higher upfront cost per capacity added.^36^ Hence, MDBs may need more budgetary room to facilitate the transition and policy debates on reforming MDB capital adequacy frameworks relate to this.^37^ In lack of budgetary room, an option may be for MDBs to advance the standardization of renewables further to lower due diligence costs.

Fourth, we observe that electricity generation investments flow disproportionately to middle-income countries, and low-income countries receive comparatively little investment as MDBs decarbonize. While we imply no causal relation, a reason for the shift away from low-income countries during decarbonization may be the high capital intensity of renewables, which makes investments in countries with comparatively worse institutional quality more costly, particularly as MDBs increasingly seek to mobilize private capital.^38^ The decentralized nature of renewables vis-à-vis fossil fuels may add to this challenge as appropriation in the case of non-payment is difficult for distributed assets. Compounding the challenge, the need for dedicated policies to change an energy sector that historically was built on fossil fuels requires institutional capacity, which may lack in many low-income countries.

Overall, we see MDBs falling short of contributing their adequate share to the expansion of renewable energy in line with PA targets. This situation calls for more focus on the institutional and systematic hurdles for renewables investment in low-income countries and a better alignment of MDB operations to the needs of these countries, which may necessitate a revision of investment and default risk frameworks to increase the risk tolerance in MDB investment decisions.^39^ Beyond the analysis of singular MDBs, the discussion on the global climate finance architecture requires more research into the interplay of MDBs with other—both established and emerging—international financial institutions, such as national development banks (both from OECD countries and from China), Export Credit Agencies, and climate finance funds with direct access such as the Green Climate Fund.^40^ As the landscape of climate finance is diversifying, it becomes ever more important to consider the full range of international financial flows. It is the sum of investments from all relevant sources that determines the future electricity generation capacity and technology mix and thereby the electricity provision and emission impacts of EMDCs.

## Methods

We analyze the operations of 10 MDBs and their subsidiaries. This includes the globally active WBG; the regional development banks AfDB, AsDB, IDB, EIB, and EBRD; as well as the so-called South-South development banks including the CAF and IsDB. For more recent years, the analysis also includes the activities of two new MDBs, the NDB and the AIIB, founded in 2014 and 2015, respectively.^13^ We note that analyzing South-South banks comes with a caveat that data availability is particularly low and restricted.

### Investment data

Our analysis covers all MDB investments in electricity generation from 2006 to 2020 reported in the MDBs’ respective online databases. We impose three important constraints. First, we explicitly exclude investments in energy storage, distribution, transmission projects, or energy efficiency measures. We exclude these components because our focus is on generation capacity, which typically precedes and drives the need for investments in storage, distribution, and transmission. Second, we limit our scope to investments considered as development finance—i.e., only to non-high-income recipients as defined by World Bank classifications^41^—to focus on the MDBs’ primary investment targets being EMDCs (e.g., EIB operations in Central Europe are excluded). Third, we exclude guarantees, resulting in a sample of 1,230 projects in the period 2006–2020.

The article is based on a newly extended database of MDB financing in electricity generation technologies, initially developed by Steffen and Schmidt^13^ and following their approach. The “bottom-up” compilation of the database aims to be exhaustive for all electricity generation projects and portfolios of the named MDBs from 2006 to 2020. The database contains projects that made it past the approval stage. It avoids counting of non-generation activities or double counting by manual analysis of each financing commitment and, if necessary, separating them from non-generation activities (such as electricity transmission, capacity building) or splitting the instrument amount by responsible co-financing institutions (e.g., multiple MDBs). We focus on financing commitments, in line with the MDB joint climate finance reporting (see 2023 Joint Report on Multilateral Development Banks Climate Finance^17^). For instance, one can observe decarbonization patterns much earlier in commitment data compared with disbursement data, as disbursements often happen over several years.

For coding each project, we primarily used project facts sheets, appraisal reports, and other related documents published by the MDBs. Based on those documents, we coded name, country, technology, financing instrument, and amount. Missing data were found by searching for secondary sources through online research, mainly press reports on the projects. While for the CAF we relied on their annual reports including short summaries of their approved projects, as in Steffen and Schmidt,^13^ for the IsDB, it was now possible to use their new online project database, which we also used for the other banks, including the newly added AIIB and NDB. The coding of the projects in 2016–20 was conducted by eight researchers (co-authors and research assistants). Each entry was checked by a second coder. For the projects from 2006–2015, some new projects that only appeared after 2017 in the MDB online databases were added. This was necessary, as some projects are only published by the MDBs months, sometimes even years, after appraisal. As a consequence, our dataset contains minimal deviations from the data in Steffen and Schmidt (2019).^13^

For each investment, we record the MDB branch, the financing instrument, the technology, and the recipient country. Namely, first, MDBs’ financing activities cover public as well as private sector projects. Some MDB branches only serve private sector clients (IFC, MIGA, and IDB Invest); other banks (EBRD, AfDB, NDB, and AIIB) indicate in their project data sheets whether it is private or public; and for all remaining projects, the status can be assigned according to the financing recipient (government entities, including SOEs, vs. private companies). Based on this information, the projects were coded as public, private, or mixed/unspecified (i.e., public-private partnerships or missing data).

Second, single MDB projects sometimes include several financing instruments (e.g., partial grant, loan, and guarantee) or multiple countries (e.g., a framework that can be accessed by more than one country in a region). To accurately represent instrument types and country allocation, we split single MDB projects into sub-items. These can be (re-)aggregated and thus ensure correct calculation of average project size, i.e., financial volume. For 24% of financial volume (2006–20), we split a project into multiple sub-items, in case there is more than one instrument type (grant, loan, guarantee…) and/or there is more than one country that receives money. This allows us to filter out guarantees or to correctly allocate the money to the receiving country. When counting projects or estimating project size, we aggregate all sub-items (cumulating the instrument amount over instrument type and multiple countries), e.g., over their different financing instruments (50 million USD_2020_ loan + 10 million USD_2020_ grant add up to 60 million USD_2020_ project size) or over different countries (a portfolio/framework covering 10 pacific islands with 2 million USD_2020_ loan each would accumulate to one project of size 20 million USD_2020_).

Third, several projects include multiple types of renewable energy and do not provide a technology split. This can lead to mixed hydro and other renewable projects ending up in the “unspecified” category, although 100% of the investment is in renewables. This represents USD_2020_ 2.6 billion (∼1.9%) of overall investment. Fourth, to allocate financing commitments to the countries that received the funding, we classified projects into six different categories and treated them as shown in Table S2. For geographical figures and figures by income groups, we only use categories 1–4, as country-specific commitment allocation is not possible for the majority of multi-country projects. These categories cover 97% of finance commitments. We group countries into income levels based on historical yearly classification provided by the World Bank.^41^

All financing commitments are presented in 2020 USD. For the conversion of local currencies, we used yearly exchange rates from the IMF’s Financial Statistics (if no USD amount was given in the project data sheet). For conversion to real USD_2020_, we used the US Consumer Price Index from the IMF’s International Financial Statistics.^42^ Note that we always recorded the financing commitment, irrespective of the associated capacity addition. This means that the investment numbers may contain refinancing, although refinancing accounts for a small share of MDB activities due to their mandates.^43^

### Policy data

The MDB policy analysis in this article is based on a newly compiled dataset. According to the Cambridge Dictionary, a policy “is a set of ideas, or a plan of what to do in particular situations, that has been agreed officially by a group of people, a business organization, a government, or a political party.”^44^ Based on this definition, we adopted the following criteria for identifying policies to be included in our analysis:

(1) Compliance with the policy definition:
  (a) targeted at achieving an intended outcome, and
  (b) official agreement of the MDB’s governance body.
(2) Focus on climate change mitigation in general, or energy specifically.
(3) Global or regional scope.

According to the quoted definition, a “policy” is always targeted at achieving an outcome, thereby differentiating itself from other forms of statements such as descriptive reports or position papers without any recommendation. Furthermore, the acting entity—in our case mostly an MDB’s board of directors—must have officially agreed on the policy. Therefore, a policy is only included in our database if the official policy document is available, not just a news article or press release. Regarding the topical focus of our analysis, we are exclusively interested in climate change mitigation policies with potential influence on electricity generation. Furthermore, we include those policies that are not just generally mitigation-related but are explicitly directed toward energy finance. The global or regional policy scope implies that the policy must target more than one specific country. As our analysis is on the level of overall MDB governance effectiveness, we look for policies with a broader scope.

The database for MDB policies between 1990 and 2020 has been compiled via manual searches on MDB websites and their online archives. More precisely, policies have been identified via Boolean searches of combinations of keywords related to sustainable electricity generation, concretely: energy AND (renewable OR environment OR climate change OR sustainable). We expect these combinations of keywords to be stable indicators of strategic alignment toward renewable energy technologies over the 1990–2020 period. Each policy has only been counted once and is attributed to the year of its introduction. If a policy has been revised substantively afterward, the updated version has been counted as a new policy.

The policy data are based on qualitative text analyses. As there is a variety of policies that are partly very heterogeneous, any comparisons between the banks or analyses of effectiveness require classification. Literature on policy instruments has put forth many taxonomies that aim at classifying policies in a mutually exclusive and collectively exhaustive way.^31^ However, these existing approaches from the field of public policy proved to be insufficient for our case; they are usually focused on government policies concerning the relationship between the state and individuals. MDB governance has a different scope, where some of these categories are not applicable. For instance, following Christopher Hood’s resource-based policy classification approach, “exercising authority” plays an important role in several coding schemes.^45^ This does not apply to MDBs, as they do not have the mandate for regulatory activities toward individuals or companies. Other Hood-categories are too broad to generate useful insights; “treasury,” for example, covers all sorts of payment instruments and thereby many of the most important MDB policies, as the banks mainly act by providing or not providing financing. However, if we summarize them in this way, we lose important information, such as the type of financing mechanism. Similar mismatches of policy scope occur when trying to approach the classification with taxonomies from the field of corporate governance. The unique institutional design of MDBs,^46^ therefore, requires a more inductive approach. The classification method we developed is derived from the one described by Howlett,^31^ concentrating on the mode of action, i.e., the mechanism that causes the desired effect. Wenzelburger,^47^ for instance, presents the following differentiation of five policy types: regulation-based policies, incentive-based policies, the creation of new offers, persuasion and information, as well as role models (e.g., state representatives). Although this approach does not cover all relevant MDB dimensions either, it is the most promising one, as the range of MDB policies can indeed be differentiated based on their modes of action. Therefore, we adopt the logic of this classification but develop the categories in an inductive way, following a mixture of a bottom-up approach and a structure introduced by Hachem et al.^48^

The nine distinct policy types clustered in five categories are listed in Table S3. The strategy category summarizes all activities that formalize the MDBs’ plans for climate-related goals and strategies to reach them. Financing climate action is resource-oriented; it encompasses all activities providing new earmarked financing for specific project types. The lending policy category covers policies that are targeted at a bank’s lending processes, i.e., all binding standards and guidelines that direct MDB financing to low-carbon projects. Internal processes are also process-oriented; however, they are non-binding and, in general, targeted at building internal capacities that will indirectly lead to financing low-carbon projects. The final category of capacity building aims at enabling long-term overall system change; it encompasses all external activities that do not directly lead to new climate-related investments but have the long-term effect of increasing demand for low-carbon projects.

When classification proved not to be mutually exclusive, all encompassed policies were counted. That means that when we found a policy that encompassed two policy types, e.g., a strategic document that also contains an emission reduction goal, it was counted for both policies individually. This makes sense, as banks have different “rhetorical” and strategic ways of publishing their policies. Comparing them, however, requires a breakdown of all governance mechanisms.

To validate the accuracy and comprehensiveness of our approach, as well as the clarifying questions on specific identified policies, we use a series of semi-structured interviews. Six interviews were conducted with senior officials involved in energy financing activities at six different MDBs. Two additional interviews were conducted with researchers in academia and a think tank to cover the new MDBs. CAF declined, and IsDB did not respond to the interview request. The interviewees were identified via public contact data and the authors’ networks. All interviews were held under the Chatham House Rule, which is why no references can be made to specific interviewees and their affiliations. Instead, Table S4 provides an overview of interviewees’ roles. Interviewees confirmed that we identified the most important policies. The five categories were refined considering the insights from the interviews.

## Resource availability

### Lead contact

Further information and requests for resources should be directed to and will be fulfilled by the lead contact, Florian Egli (florian.egli@tum.de).

### Materials availability

This research did not generate any new materials.

### Data and code availability

The two used datasets on investments and policies of MDBs and the visualization code to reproduce all figures are separately available on Zenodo under the following link: https://doi.org/10.5281/zenodo.17158311.

## Acknowledgments

The authors are grateful for research support from David Grivel, Nielja Knecht, Rui Zhang, Srihari Srivathsan, and Victor Hopo and for feedback from participants at the 2024 International Symposium on Climate, Finance, and Sustainability in Paris. This work benefitted from the European Union’s Horizon 2020 research and innovation program, European Research Council (ERC), under grant agreement no.948220 (N.P. and B.S.), and it was supported by the Swiss State Secretariat for Education, Research and Innovation (SERI) under contract no. 24.00550, as part of the European Union’s Horizon Europe research and innovation program project NEWPATHWAYS (B.S.). The opinions expressed and arguments employed herein do not necessarily reflect the official views of the European Commission or the Swiss Government.

## Author contributions

F.E., T.S.S., and B.S. conceptualized and planned the research. N.P. collected data; F.E., N.P., and C.-M.L. analyzed the data; and C.-M.L. visualized it. F.E., N.P., and C.-M.L. wrote the initial draft; B.S., T.B., and T.S.S. provided input, commented, and edited the draft.

## Declaration of interests

The authors declare no competing interests.

## Supplemental information

Document S1. Figures S1–S6 and Tables S1–S4

Document S2. Article plus supplemental information
