# Supplementary material for: Tracking decarbonization of multilateral development banks’ electricity generation investments
Source: Cell Rep Sustain. 2025 Dec 26;2(12):100584. doi: 10.1016/j.crsus.2025.100584 (PMC12743014; doi:10.1016/j.crsus.2025.100584)
Supplement: Document S1. Figures S1–S6 and Tables S1–S4 [file mmc1.pdf]

CRSUS, Volume *2*

Supplemental information

Tracking decarbonization of multilateral development banks' electricity generation investments

Florian Egli, Clemens-Maria Lehofer, Nadine Palmowski, Tim Büthe, Bjarne Steffen, and Tobias S. Schmidt

Supplemental Information

Supplemental Figures

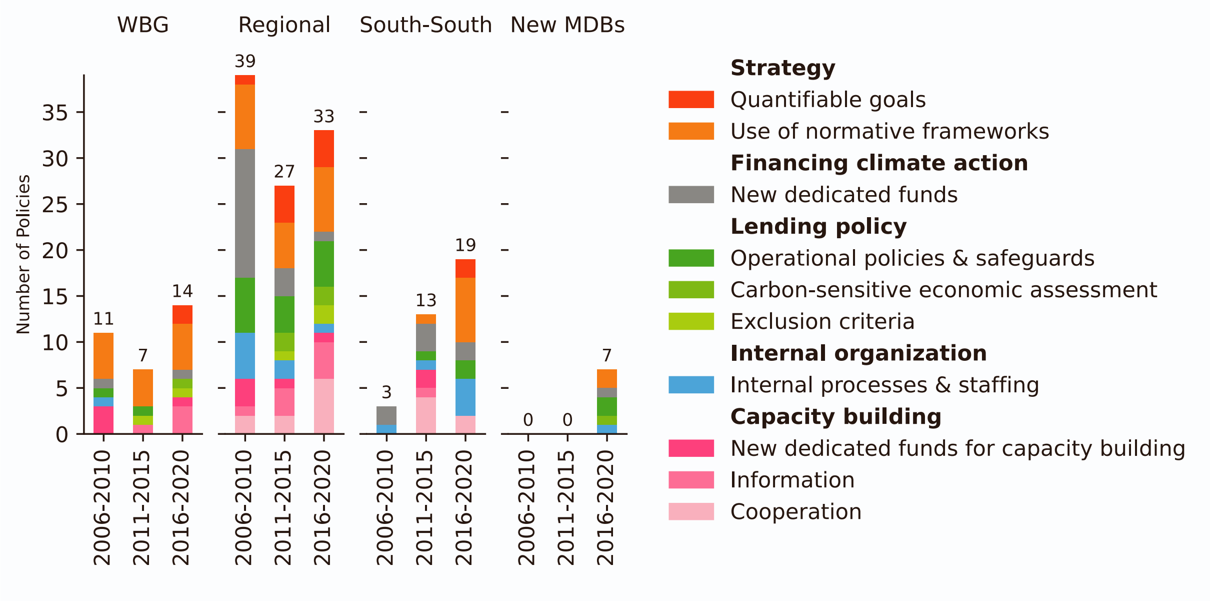

Figure S1. Number of policies per MDB category in the respective time period.

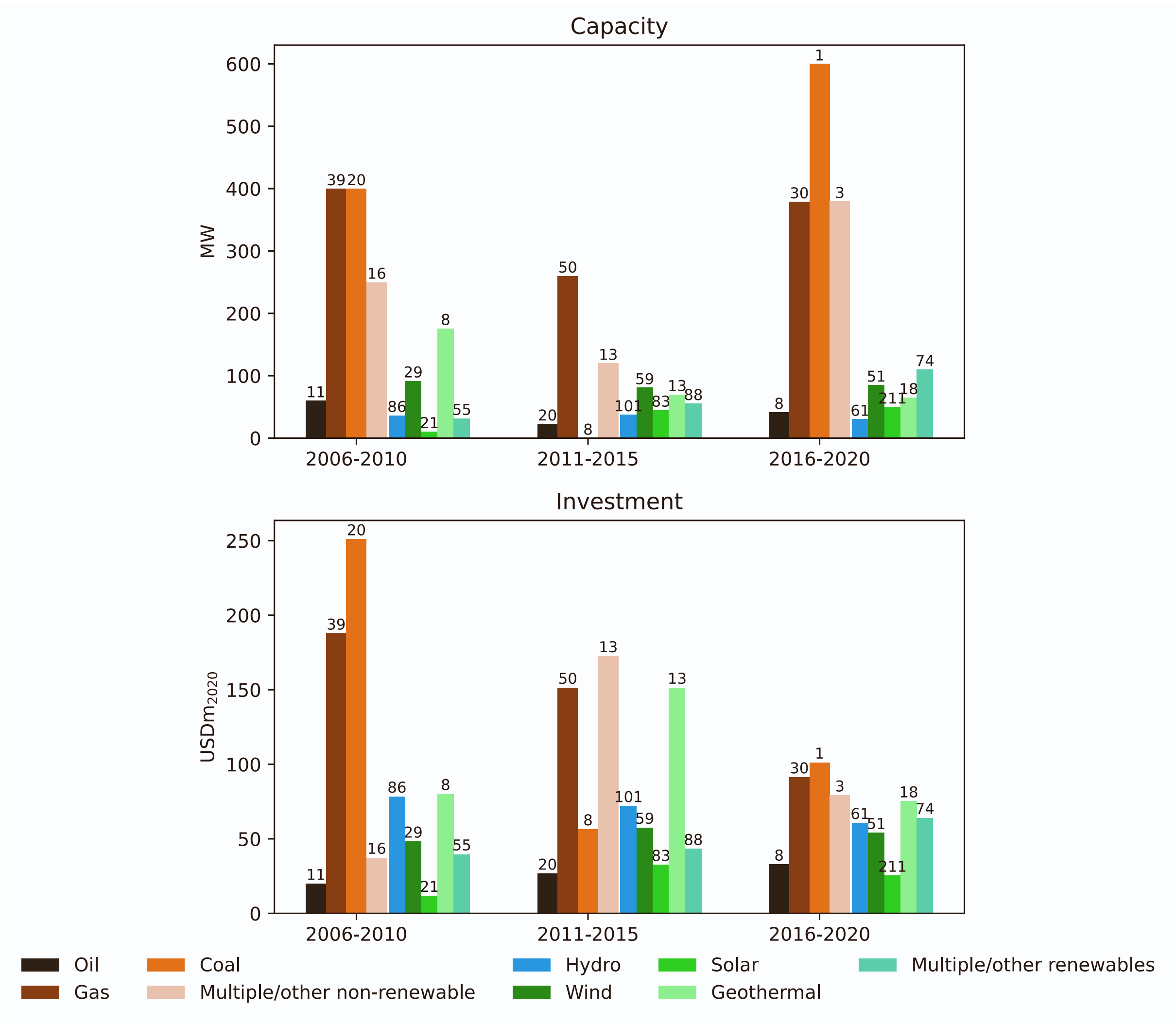

Figure S2. Median project size (investments and capacity added) per technology in respective time periods.

Excluding guarantees and investments in high-income countries.

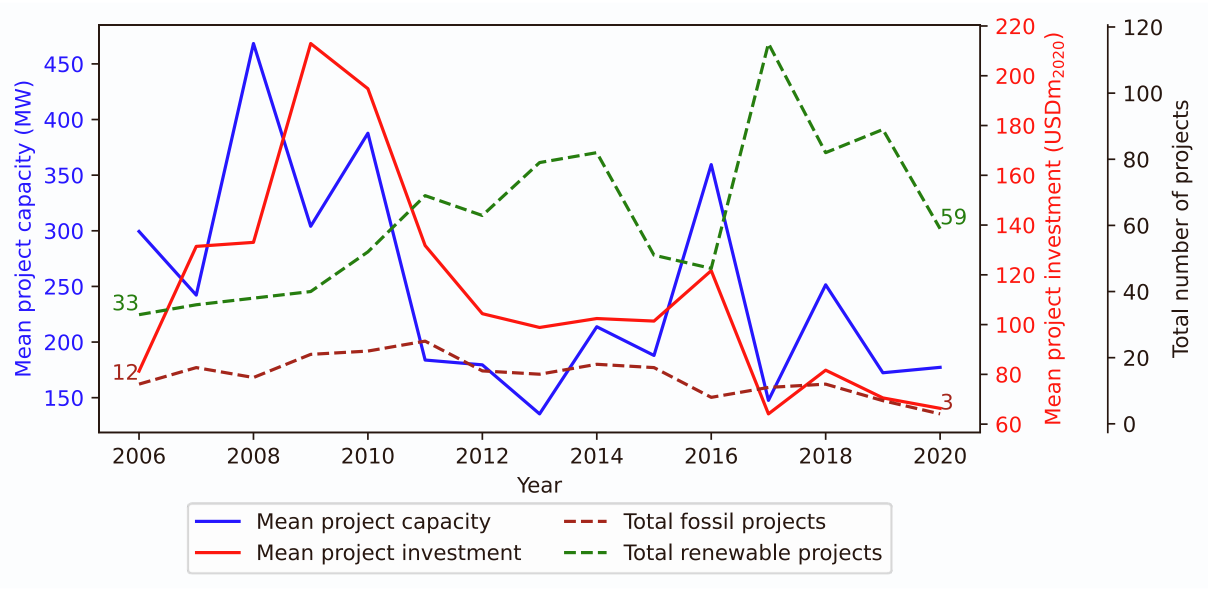

Figure S3. Average project size (funds committed and capacity added) and total number of projects by technology group over time.
Excluding guarantees and investments in high-income countries.

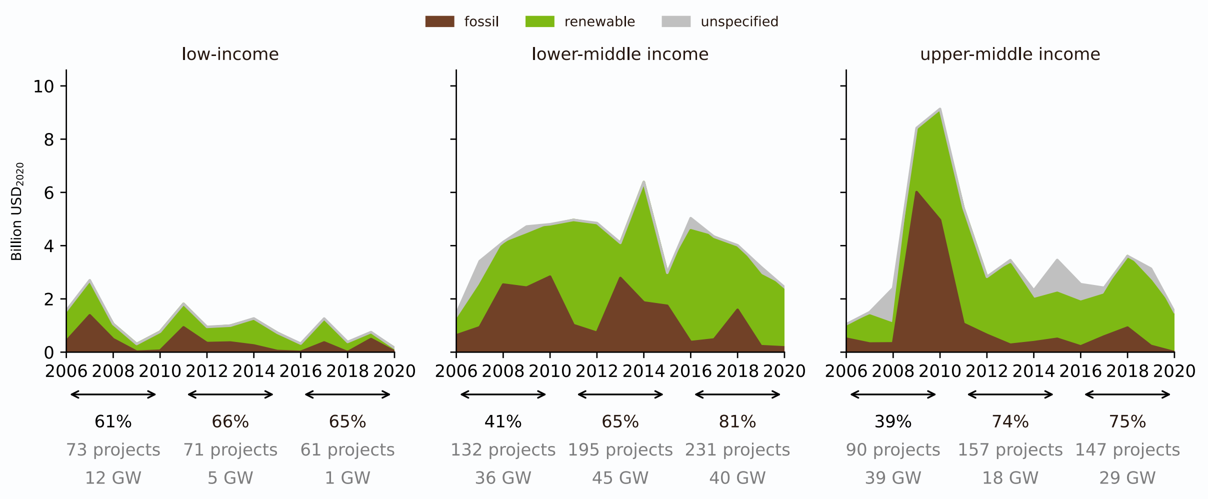

Figure S4. Yearly MDB investment per technology and recipient income group.
Country classification follows the historic World Bank classifications, i.e. countries may move across groups over the observed time period. Sub-figure numbers indicate the share of renewables, the total number of projects as well as the total capacity added within the respective time period, i.e. 2006-2010, 2011-2015, 2016-2020. Figure data excludes guarantees and investments in high-income countries, as well as multi-country projects, where recipient countries could not be identified (see Methods).

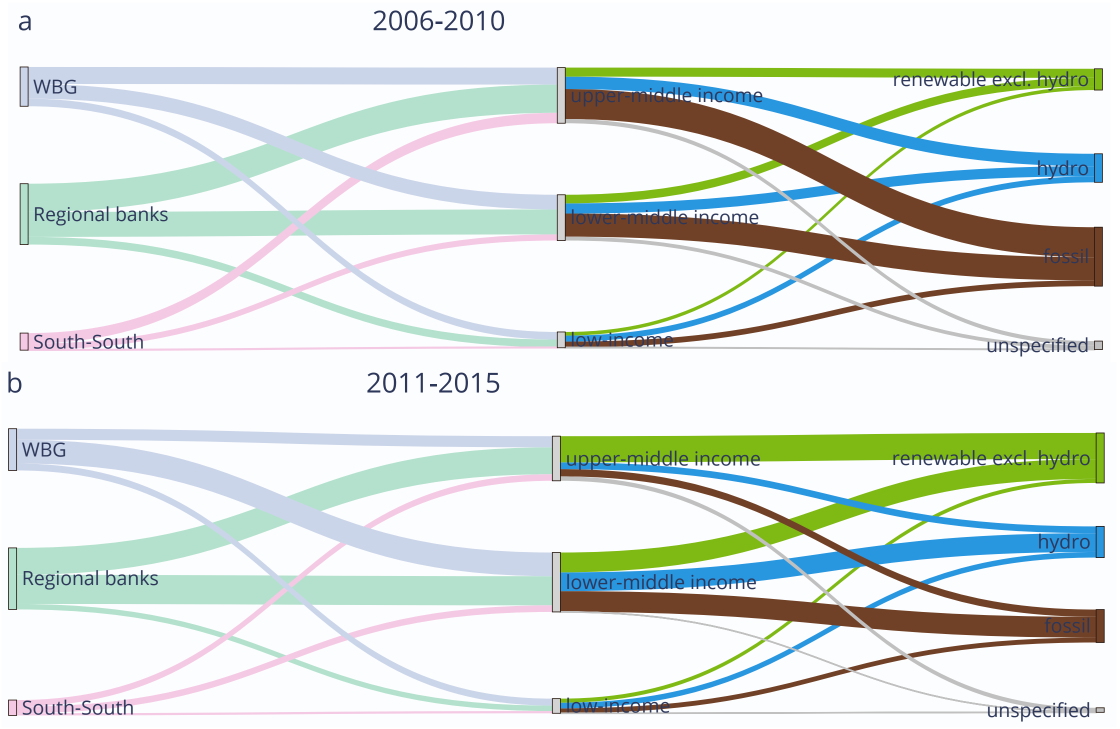

Figure S5. Total investment flows to country income groups and technology group for (a) 2006-2010 and (b) 2011-2015.
Excluding guarantees and investments in high-income countries.

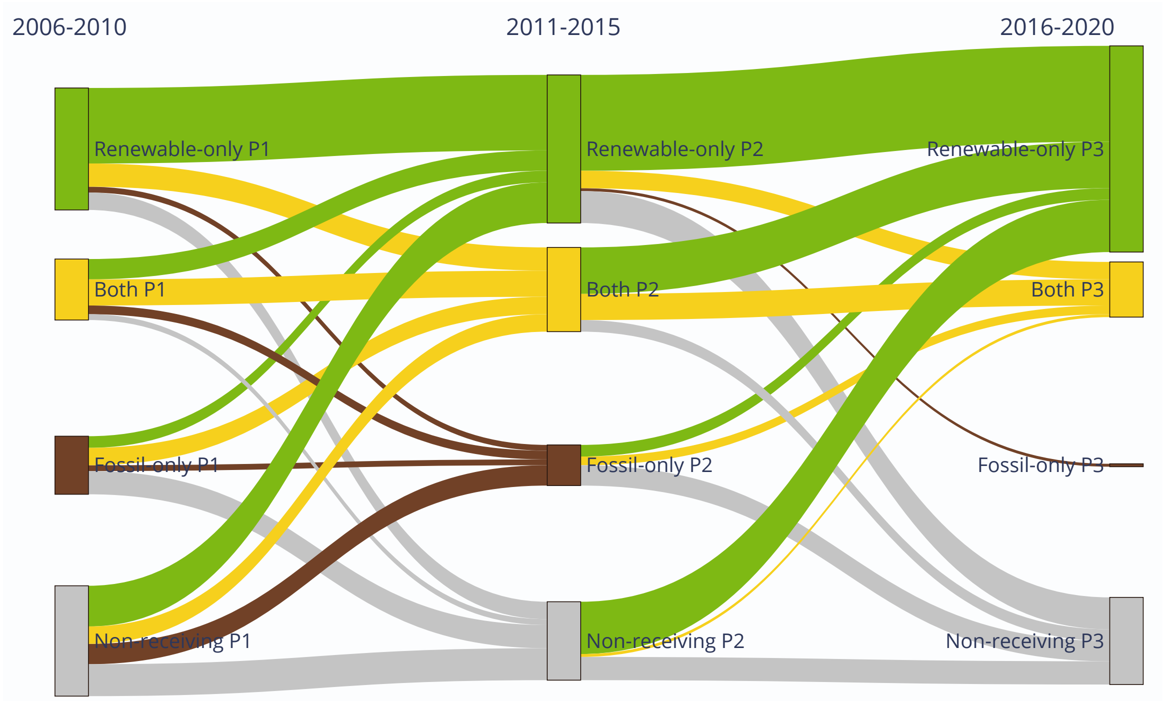

Figure S6. Movement of countries across investment groups between time periods.
P1 = 2006-2010, P2 = 2011-2015, P3 = 2016-2020. Investment groups include renewable-only, fossil-only, both and non-receiving recipient countries. Excluding guarantees and investments in high-income countries.

Supplemental Tables

Country	2006-2010 Total Investment (USDm)	2016-2020 Total Investment (USDm)	Absolute Change (USDm)	Relative Change (%)
Rwanda	5.43	66.67	61.24	1128
Afghanistan	14.42	129.36	114.93	797
Chad	3.11	20.75	17.63	566
Central African Rep.	9.65	49.83	40.18	416
Mozambique	94.95	484.61	389.66	410
Guinea	9.24	34.63	25.39	275
Burkina Faso	48.43	180.28	131.85	272
Mali	140.01	327.38	187.37	134
Madagascar	53.51	96.77	43.26	81
Burundi	78.38	81.00	2.62	3
Liberia	0.00	58.23	58.23	-
Niger	0.00	52.68	52.68	-
Malawi	0.00	30.37	30.37	-
Somalia	0.00	5.93	5.93	-
The Gambia	0.00	103.95	103.95	-
Sum of positive abs. change			1265.29	
Uganda	944.54	309.36	-635.18	-67
Ethiopia	162.27	11.60	-150.67	-93
Democratic Republic of the Congo	564.83	32.15	-532.68	-94
Guinea-Bissau	15.07	0.00	-15.07	-100
Togo	16.89	0.00	-16.89	-100
Sum of negative abs. change			-1350.50	

Table S1. Aggregated investment ($USDm_{2020}$) of countries that have been low-income for the full sample period (2006-2020) for 2006-2010 and 2016-2020 and the respective absolute and relative changes between the two periods.

Number	Category	Share of commitments	Note
1	Electricity generation assets in one country	80.4%	*Commitments allocated to the one single country according to the data source.*
2	Electricity generation assets with multiple countries as beneficiaries	0.7%	*We split equally between benefiting countries, if no further indication in MDB project sheet*
3	Portfolio/framework for a dedicated country	15.0%	*Can be allocated to the respective country according to the data source.*
4	Balance sheet category (one country)	1.3%	-
5	Portfolio/ framework for multiple countries/regions	2.4%	*Such commitments are lacking an indication on split of actual money spent (e.g., a framework is provided for 10 pacific islands, but there is no information on the split). We are excluding these projects from the geographical analysis.*
6	Balance sheet category (multiple countries)	~0.1%	-

Table S2. Country allocation of financial commitments. Share of commitments excludes guarantees.

Category	Sub-category	Example
Strategy	Quantifiable goals	- Target for relative share of climate finance or RE
	Use of normative frameworks	- Action plan, bank-wide climate strategy
Financing climate action	New dedicated funds	- Earmarked financing for specific type of project / technology
Lending policy	Operational policies and safeguards	- Environmental standards
	Carbon-sensitive economic assessment	- Shadow pricing
	Exclusion criteria	- Excluding coal investment excluding upstream oil & gas
Internal processes	Internal processes and staffing	- Hiring climate experts, setting up a climate task force, climate mainstreaming, training or staff
Capacity building	New dedicated funds for capacity building	- Funds for technical assistance
	Information	- Setting up a database for best practice sharing
	Cooperation	- Joining international alliance, e.g. Task Force on Climate-Related Financial Disclosures (TCFD)

Table S3. Policy type categories with subcategories and examples.

ID	Institution	Role of interviewee	Status	Format	Length	Recording	Transcript
1	MDB	Energy sector specialist	Conducted on 10.05.21	Semi-structured	1 hour	Concurrent notes	Confidentiality requested
2	MDB	Board member (for creditor country)	Conducted on 02.06.21	Semi-structured	1 hour	Concurrent notes	Confidentiality requested
3	MDB	Financial markets specialists	Conducted on 28.06.21	Semi-structured	1 hour	Concurrent notes	Confidentiality requested
4	MDB	Senior energy economist	Conducted on 02.07.21	Semi-structured	1 hour	Concurrent notes	Confidentiality requested
5	MDB	Principal banker	Conducted on 19.07.21	Semi-structured	1 hour	Concurrent notes	Confidentiality requested
6	MDB	Division manager energy	Conducted on 30.07.21	Semi-structured	1 hour	Concurrent notes	Confidentiality requested
7	Research	Senior researcher on MDBs	Conducted on 28.06.21	Semi-structured	1 hour	Concurrent notes	Confidentiality requested
8	Think tank	Senior researchers on Energy and Climate	Conducted on 03.06.21	Semi-structured	1 hour	Concurrent notes	Confidentiality requested

Table S4. Interview Methods Table (as in ref. [S1]). Overview of interview dates and method, and interviewees' role.

Supplemental References

[S1]. Bleich, E., and Pekkanen, R. (2015). Data Access, Research Transparency, And Interviews; The Interview Methods Appendix. https://doi.org/10.5281/ZENODO.892386.
